# Comparative Enzymology and Biomass Hydrolysis Reveal Industrial Biorefining Potential of *Aspergillus fumigatus* Strain VP2T

**DOI:** 10.3390/microorganisms14030723

**Published:** 2026-03-23

**Authors:** Vaniksha Pal, Punam Vishwakarma, Dipayan Samanta, Priya Saxena, Rohit Rai, Rajesh K. Sani

**Affiliations:** 1 Department of Microbiology, Lovely Professional University, Phagwara 144411, India; vaniksha11919618@gmail.com; 2 Faculty of Applied Medical Sciences, Lovely Professional University, Phagwara 144411, India; punamvishwakarma0812@gmail.com; 3 Department of Chemical and Biological Engineering, South Dakota School of Mines and Technology, Rapid City, SD 57701, USA; dipayan.samanta@sdsmt.edu (D.S.); priya.saxena@sdsmt.edu (P.S.)

**Keywords:** *Aspergillus fumigatus*, lignocellulose, crystalline cellulose deconstruction, biomass conversion, lytic polysaccharide monooxygenase (LPMO), redox synergy

## Abstract

We report on the isolation and comprehensive genomic and biochemical characterization of *Aspergillus fumigatus* VP2T, a thermophilic filamentous fungus recovered from Himalayan Forest soil with exceptional lignocellulolytic capacity. Whole-genome sequencing revealed a 32.1 Mb genome encoding 12,675 predicted genes, including an extensive repertoire of >300 carbohydrate-active enzymes (CAZymes). Notably, the genome harbors multiple auxiliary activity enzymes, including AA9-family lytic polysaccharide monooxygenases and several cellobiose dehydrogenases (CDHs), supporting oxidative–hydrolytic synergism during biomass degradation. Submerged fermentation using a cellulose–wheat bran–rice straw substrate induced high enzyme titers, including 33 U/mL endoglucanase and 131 U/mL CDH, exceeding activities commonly reported for both native and engineered fungal strains. Although exoglucanase (0.02 U/mL) and xylanase (14.22 U/mL) activities were comparatively modest, the strain VP2T demonstrated superior hydrolysis of untreated rice straw, achieving a 1.89-fold increase in saccharification efficiency relative to the commercial enzyme cocktail Cellic^®^ CTec2. Scanning electron microscopy confirmed extensive disruption of lignocellulosic architecture, consistent with enhanced enzyme accessibility and oxidative fiber loosening. Collectively, genomic evidence and functional assays identify *A. fumigatus* VP2T as a redox-optimized, moderately thermophilic biocatalyst suited for low-pH lignocellulose conversion. This study highlights the value of exploring thermophilic fungal biodiversity to discover native strains with inherent oxidative capacity, offering promising alternatives to pretreatment-intensive biorefinery processes and informing the rational development of tailored enzyme systems.

## 1. Introduction

The efficient and sustainable valorization of lignocellulosic biomass (LCB) into renewable fuels and high-value biochemicals is a cornerstone of next-generation green bioeconomy development. Composed mainly of cellulose, hemicellulose, and lignin, LCB is a renewable, non-food-based resource abundantly available from agricultural residues and forestry byproducts [1]. However, its heterogeneous, compact, and highly recalcitrant structure limits its enzymatic digestibility, thus posing major techno-economic hurdles for second-generation biorefineries [2]. Overcoming the bottlenecks of efficient biomass deconstruction without harsh pretreatments is key to unlocking its potential as a sustainable feedstock.

Current industrial strategies rely heavily on commercial enzyme cocktails, such as Cellic CTec (Novozymes), DuPont Accellerase, and DSM’s advanced blends [3,4]. These enzyme systems primarily consist of endoglucanases (EGs), cellobiohydrolases (CBHs), and β-glucosidases (BGLs), which work synergistically to depolymerize cellulose into fermentable sugars [5]. Yet, these formulations are limited by their high production costs, temperature sensitivity, poor performance on untreated substrates, and a general lack of oxidative enzymes such as lytic polysaccharide monooxygenases (LPMOs) and cellobiose dehydrogenases (CDHs), which have been shown to play essential roles in crystalline cellulose degradation [6,7,8,9]. Moreover, the requirement of costly chemical or thermal pretreatments not only raises operational costs but also results in the formation of inhibitory compounds, further limiting the practical scalability of bioconversion platforms [10].

Filamentous fungi, particularly species from *Aspergillus* and *Trichoderma*, are considered natural lignocellulose degraders and serve as prolific sources of hydrolytic enzymes [11]. Among them, *Aspergillus fumigatus* has emerged as a promising candidate due to its thermostable enzymes, metabolic versatility, and capacity to secrete a broad range of glycoside hydrolases and accessory enzymes, including those with oxidative capabilities [12,13]. However, most studies have focused on recombinant or genetically optimized strains, often overlooking wild-type isolates that have naturally evolved enzymatic arsenals tailored for survival in complex ecological niches [14]. These wild strains may harbour novel enzymatic combinations or regulatory mechanisms that can surpass the efficiency of engineered systems, especially under industrially relevant, low-input conditions [15].

Recent insights into fungal biomass degradation have revealed the critical role of oxidative enzymes such as LPMOs (AA9 family) and CDHs, which cleave polysaccharide chains through redox reactions, improving the accessibility of hydrolytic enzymes to crystalline cellulose regions [16]. The synergistic action between CDH and LPMO systems significantly enhances saccharification yields, especially in raw, untreated biomass, offering an attractive alternative to conventional strategies [17,18]. Despite this, commercial formulations still lack robust representation of such oxidative enzymes, further reinforcing the need to explore native fungal systems with high redox synergy.

Therefore, the objective of this study was to isolate and characterize a thermophilic fungal strain, *Aspergillus fumigatus* VP2T, and to investigate its lignocellulolytic enzyme system through integrated genomic and enzymatic analyses. Furthermore, the study aims to evaluate the biomass hydrolysis potential of the VP2T secretome and assess its suitability for industrial lignocellulosic biorefinery applications. This study addresses critical gaps in fungal lignocellulose deconstruction by isolating and characterizing a novel thermophilic wild-type strain, *Aspergillus fumigatus* VP2T, from forest soils in the Himalayan region of Uttarakhand, India. The strain demonstrated a strong lignocellulolytic profile, notably secreting both hydrolytic and oxidative enzymes under submerged fermentation conditions. Preliminary screening revealed its ability to hydrolyze untreated biomass more efficiently than commercial enzyme blend Cellic CTec2, suggesting the presence of a naturally optimized, redox-driven enzymatic system. To understand the genetic basis of this capability, whole genome sequencing and computational analyses were performed, focusing on gene prediction, repeat elements, and annotation of carbohydrate-active enzymes (CAZymes). The genome analysis revealed a diverse enzymatic repertoire, including key glycoside hydrolases and auxiliary activity enzymes such as LPMOs and CDH. By integrating enzymology with genome-level insights, this study establishes *A. fumigatus* VP2T as a promising candidate for pretreatment-free biomass bioconversion and lays the foundation for future development of robust fungal enzyme systems for sustainable biorefineries.

## 2. Materials and Methods

### 2.1. Isolation and Optimization of Growth Conditions for the Thermophilic, Lignocellulolytic Fungal Isolate VP2T

A thermophilic, lignocellulolytic fungus designated as VP2T was isolated from soil samples collected at Ranikhet forest range, Uttarakhand, India (29°39′ N 79°25′ E). The isolation was performed on Potato Dextrose Agar (PDA; HiMedia, Thane, India) at 45 °C under a pH of 5.6 for 5 days. To prevent bacterial contamination, ampicillin (100 µg/mL) was incorporated into the medium. To ensure purity, the strain underwent successive sub-culturing on PDA, and the purified culture was stored on agar stubs at 4 °C for short-term maintenance and preserved at −80 °C in 25% glycerol for long-term storage. To determine the optimal growth conditions for strain VP2T, several factors were systematically evaluated. These included the choice of cultivation media (Potato Dextrose Agar, Czapek Yeast Extract Agar, Yeast Phosphate Soluble Starch Agar, and Oatmeal Agar), temperature range (35–55 °C), pH range (4.0–8.0), and incubation time (3–7 days) [19,20,21,22].

### 2.2. A Combined Morphological and Molecular Characterization of the Thermophilic Isolate VP2T

A combined morphological and molecular approach furnishes robust insights into both the phenotypic and genotypic traits of the isolates, ensuring a comprehensive characterization [23]. Morphological studies of the isolated VP2T strain involved both macroscopic and microscopic examinations. The colonies grown on PDA were evaluated for their appearance, color, texture, and other pertinent physical attributes. In parallel, microscopic observations focusing on the structure and arrangement of phialides and spores were performed using slide culture technique [24]. Further, molecular characterization of VP2T was achieved by amplifying the 18S rDNA sequence with universal primers ITS1 (5′-CCGTAGGTGAACCTGCGG-3′) and ITS4 (5′-TCCTCCGCTTATTGATATG-3′), following the protocol detailed by Rai and his coworkers [24,25]. For this purpose, Genomic DNA of the isolated fungal strain VP2T was extracted using a fungal DNA isolation kit (HiMedia; Thane, India; Cat No. MB543) and used as template to amplify approximately 600 bp of the ITS1-5.8S-ITS2 region with universal primers. PCR amplification was performed in a 20 μL reaction containing 1 μL DNA template, 10× Buffer, 0.2 mM dNTPs, 0.5 μM each primer, and 2 U of Taq polymerase (R001C TaKaRa Taq™, Takara Bio Inc., Kusatsu, Japan). Cycling conditions included initial denaturation at 95 °C for 3 min, followed by 32 cycles of denaturation (30 s at 95 °C), annealing (30 s at 50 °C), and extension (1 min at 72 °C), with a final extension at 72 °C for 10 min. Amplicons were purified using FavorPrep™ GEL/PCR Purification Kit (Favorgen Biotech Corp., Ping Tung, Taiwan; Cat No. FAGCK 001) and sequenced bidirectionally via Sanger sequencing at Biologia Research India Pvt. Ltd. (New Delhi, India). The resulting nucleotide sequence was used as a query for NCBI-BLAST v2.17.0 analysis against the NCBI non-redundant (nr) database using the BLOSUM62 substitution matrix to identify highly similar sequences. The top-matching sequences (11) were retrieved in FASTA format, manually curated, and compiled into a single FASTA file. Multiple sequence alignment was performed using the CLUSTAL W algorithm, and a phylogenetic tree was constructed using the Neighbor-Joining (NJ) method implemented in MEGA v11.0, following the approach described by Rai and his coworkers [23].

### 2.3. Whole Genome Sequencing of Aspergillus fumigatus Strain VP2T

The genome of *Aspergillus fumigatus* strain VP2T was sequenced using state-of-the-art next-generation sequencing technology to ensure high-quality data acquisition and comprehensive genome coverage. The Illumina HiSeq 4000 platform was utilized for its capacity to generate a vast number of paired-end (PE) reads with high accuracy and efficiency [26]. A PE library was constructed with an average insert size of 500 base pairs (bp) following the standard Illumina library preparation protocol, which included DNA fragmentation, adapter ligation, and PCR amplification. The sequencing was performed with a read length of 2 × 151 bp, generating a total of 2.91 Gb of clean data. To process the raw sequencing reads, stringent quality control steps were implemented using FastQC (v0.11.9) to assess read quality, followed by trimming and filtering with Trimmomatic (v0.39) [27,28]. This ensured the removal of low-quality reads, adapter contamination, and sequencing artifacts. High-quality reads were subsequently used for downstream analysis. The de novo genome assembly was performed using the MaSuRCA assembler (v4.1.0), which is optimized for processing large and complex fungal genomes [29]. MaSuRCA employs a hybrid approach combining de Bruijn graph and overlap-layout-consensus methods to produce high-contiguity assemblies [30]. Assembly quality metrics, including N50 scaffold length and L50 scaffold count, were calculated using QUAST (v5.0.2) [31]. The final assembly consisted of scaffolds and supercontigs systematically arranged to minimize gaps and ensure sequencing integrity. For repeat sequence identification, RepeatMasker (v4.1.1) and RepeatModeler (v2.0.1) were employed [32]. These tools systematically identified interspersed repetitive elements, including retroelements and DNA transposons, as well as simple repeats [33]. Repeat identification relied on a comprehensive repeat library, including fungal-specific elements, to provide accurate annotation of repetitive regions in the genome. The GC content of the assembled genome was calculated using SeqKit (v2.2.0), providing insights into genome composition [34]. Additionally, sequence gaps and assembly completeness were evaluated to ensure the assembly met the standards for downstream analyses.

### 2.4. Production and Screening of Lignocellulolytic Glycosyl Hydrolases by Isolated Thermophilic Fungal Strain VP2T

Shake flask (submerged fermentation; SmF) experiments were conducted to produce lignocellulolytic glycosyl hydrolases (GHs), including cellulases, auxiliary enzymes, and xylanases from the isolated thermophilic fungal strain VP2T. The experiments were performed in 250 mL Erlenmeyer flasks containing 50 mL of a cellulose (3% *w*/*v*), wheat bran (1% *w*/*v*), and rice straw (1% *w*/*v*) based (CWR) medium (pH 5.0), following the protocol described by Rai et al. [25]. Each flask was inoculated with four agar plugs of 6 mm diameter excised from the periphery of the actively growing 5-day-old culture (PDA) and incubated at 45 °C under shaking conditions (180 rpm) for 7 days. Further, the cellulase production profile of the fungal isolate VP2T was assessed using the SmF setup described earlier, with 2 mL samples withdrawn at 24-hour intervals over the 10-day production period. Additionally, the activity profile of VP2T was benchmarked against that of a commercial cellulase preparation, Cellic CTec2, at the same protein concentration of 0.7 mg/mL.

### 2.5. Assaying Enzyme Activities and Determining Protein Concentration

The culture extracts were clarified through centrifugation (8000× *g*; 20 min), appropriately diluted, and analyzed for an array of enzymatic activities following standard protocols [25]. Total cellulase activity (FPase) was measured using the method prescribed by Ghose [35]. Briefly, 0.5 mL of diluted enzyme was mixed with 1 mL of 50 mM sodium acetate buffer (pH 4.8) and a 1 × 6 cm Whatman No. 1 filter paper strip, incubated at 50 °C for 60 min. Avicelase enzyme activity was assessed by mixing 0.5 mL of enzyme with 0.5 mL of 4% (*w*/*v*) Avicel PH 101 in 50 mM sodium acetate buffer (pH 5.0), followed by incubation at 50 °C for 2 h [36]. For the endoglucanase (EG) and xylanase assays, 1 mL reaction mixtures were prepared by combining equal volumes of diluted enzyme with 2% carboxymethyl cellulose (CMC) for the EG assay, and 1% beechwood xylan (BWX) for the xylanase assay. The reaction mixtures were then incubated at 50 °C for 10 and 5 min for the EG and Xyl assays, respectively. All the reactions were terminated by adding 3 mL of dinitrosalicylic acid (DNS) reagent, followed by boiling at 100 °C for 10 min. The released reducing sugars were quantified spectrophotometrically at 540 nm using glucose (EG, Avicelase, FPase) and xylose (Xylanase) standards. One unit of the respective enzyme activity was defined as the amount of enzyme required to release 1 µmol of reducing sugar (glucose or xylose equivalents) per minute under assay conditions [24]. The β-glucosidase activity was assayed using 3 mM *p*-nitrophenyl-b-D-glucopyranoside (pNPG) (Sigma-Aldrich, St. Louis, MO, USA) and cellobiohydrolase (CBH) activity was assayed using 3 mM *p*-nitrophenyl-b-D-lactopyranoside (pNPL) and *p*-nitrophenyl-b-D-cellobioside (pNPC) (Sigma-Aldrich) as substrates CDH activity was determined by monitoring the reduction of 2,6-dichlorophenol indophenol (DCIP) at 520 nm [23,37]. The reaction mixture (1 mL) containing 20 mM cellobiose (100 µL), 0.3 mM DCIP (100 µL), 0.1 M sodium acetate buffer (pH 5.0; 780 µL), and enzyme (20 µL), was incubated at 50 °C for 5 min. Further, total protein content in the enzyme extracts was quantified using the standard Folin–Ciocalteu reagent method [38]. The activities were originally determined in U/mL and were later converted into U/gDS (units per gram dry substrate) for comparative analysis with the studies reporting enzyme activities in U/gDS in the literature. Each enzyme activity was measured using standard substrate-specific assays with distinct protocols and unit definitions, conducted separately on the same VP2T culture supernatant.

The formula used for conversion is given below:

(1) Activity in U/gDS = (Activity in U/mL × Total Volume in mL)/Mass of Dry Substrate in grams

### 2.6. Enzymatic Hydrolysis of Rice Straw Using VP2T Secretome and Its Comparison with Commercial Cellulase Preparation Cellic CTec2

The hydrolytic potential of secretome obtained from the isolated VP2T strain was assessed and compared with that of a high-performing commercial cellulase preparation, Cellic CTec2 (Novozymes, Karnataka, India). The substrates tested included untreated, alkali-treated, and acid-treated rice straw at a loading of 7% (*w*/*v*). Hydrolysis reactions (1 mL) were conducted in 5 mL glass vials with screw caps, using an enzyme dosage of 10 mg protein per gram of dry substrate (gDS). The reactions were carried out at 50 °C with mild agitation (150 rpm) for 72 h. Notably, all the hydrolysis reactions contained VP2T secretome only; no external electron donors or redox mediators were supplemented. The concentration of released reducing sugars was quantified using the 3,5-dinitrosalicylic acid (DNS) reagent as described in Rai and his coworkers [25].

### 2.7. Structural Analysis of Rice Straw Before and Post Hydrolysis with Secretome Derived from Thermophilic Isolate VP2T

Freshly harvested rice straw was collected from the agricultural fields in Bhogpur, a small town in Punjab, India (31.537872° N, 75.650643° E). The straw was mechanically cut into 2–3 cm segments, washed thoroughly with deionized water to remove soil residues, air-dried at 25 °C for 72 h, and ground with the help of a grinder and sieved through a 5–7 mm sieving mesh [39]. Two experimental sets were prepared: (1) untreated control samples and (2) samples treated with the secretome of the isolated fungal strain VP2T (as discussed in Section 2.5). For structural characterization, untreated and enzyme-treated rice straw samples were dehydrated through a graded ethanol series (30%, 50%, 70%, 90%, and 100% *v*/*v*), with 30-min immersion at each concentration to ensure complete water displacement (Hayat, 2000, Hayat Holding, Istanbul, Turkey). Dried samples were mounted on aluminum stubs with carbon tape (Agar Scientific, Essex, UK) and sputter-coated with a 20 nm gold layer (DII-29030SCTR; JEOL Smart Coater, JEOL Ltd., Tokyo, Japan) to enhance conductivity. Surface morphology was examined using FE-SEM (JEOL JSM-7610 FPLUS, JEOL Ltd., Tokyo, Japan) operated at 15 kV accelerating voltage and a working distance of 10 mm. Images were acquired at magnifications of 500×, 1000×, and 5000×, with three replicates per treatment group [40].

### 2.8. Statistical Analysis

All experiments reported in this study were performed in triplicate (*n* = 3). Enzyme activity data represent means ± standard error of the mean (SEM) calculated using Microsoft Excel (MS-Excel v365) as SEM = SD/√*n*, indicating precision of mean estimates. Hydrolysis performance data also report means ± SEM from biological triplicates at 5% significance level, enabling direct comparison of VP2T secretome versus Cellic CTec2 across substrates.

## 3. Results and Discussion

### 3.1. Morphological and Molecular Characterization of the Isolated Thermophilic Fungal Strain VP2T

**Figure 1:**
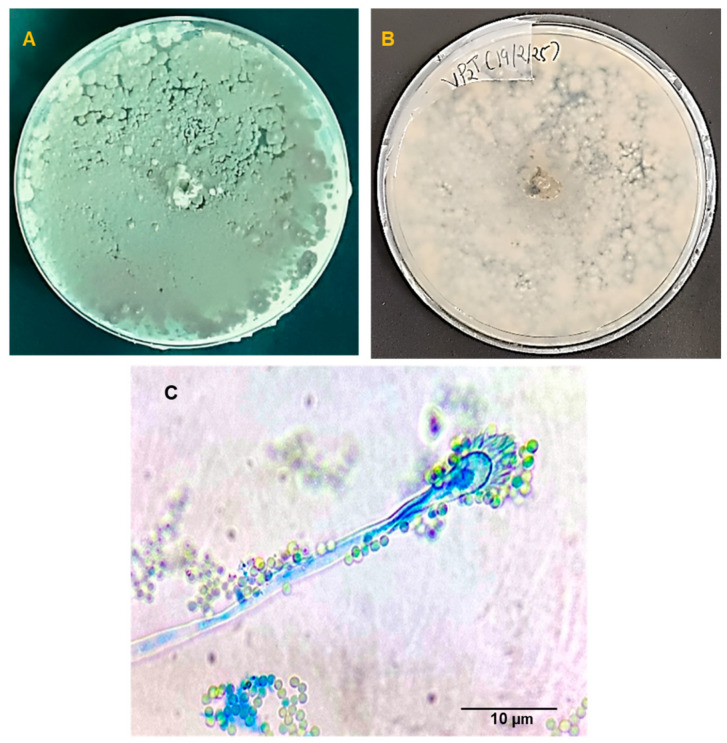
Colony growth of *A. fumigatus* strain VP2T on Potato Dextrose Agar (PDA) observed on the 7th day of incubation, showing (**A**) obverse and (**B**) reverse sides, along with (**C**) a photomicrograph depicting conidia and conidiophores at 100× magnification.

**Figure 2:**
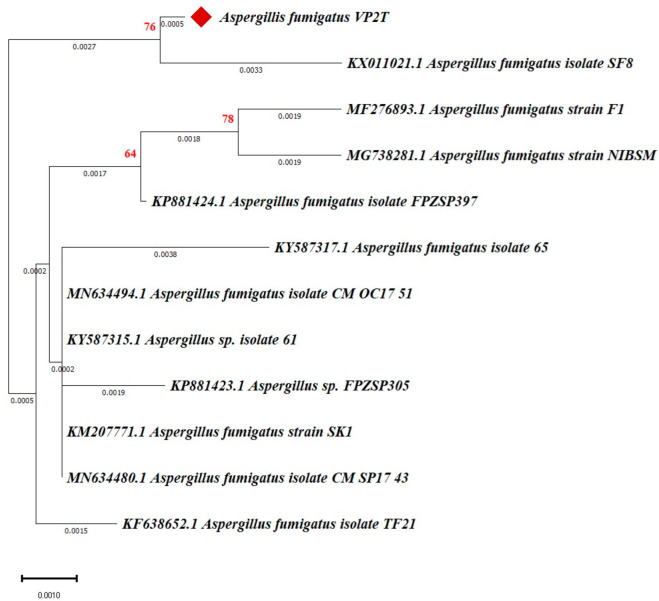
Phylogenetic tree of thermophilic fungi based on amplified ITS1-5.8S-ITS2 sequence of *A. fumigatus* strain VP2T (in red rhombus) and other related species obtained from NCBI. A consensus NJ dendrogram with bootstrap values was based on multiple sequence alignment using CLUSTALW (MEGA 11.0.13) software. Highlighted VP2T strain was used in this study. The bootstrap values are shown at nodes (>50).

Based on morphological characteristics, the thermophilic fungal isolate VP2T was identified as *Aspergillus fumigatus*. On PDA medium, the culture showed rapid growth reaching the edges of the Petri dish within 72 h at 40–50 °C, thus exhibiting the thermophilic and robust nature of the fungus that confers it the ability to thrive in various environmental conditions [41,42]. The culture produced velvety colonies with flat surfaces, exhibiting a greenish-gray color on the obverse and colorless to slightly pale-yellow color on the reverse side (Figure 1A,B). The microscopic examination of the slide culture showed septate and hyaline hyphae, forming a dense network that supported the columnar conidial heads (Figure 1C). The conidia were typically spherical to sub-spherical, exhibiting a smooth to slightly roughened surface, appearing in chains on uniseriate phialides that are borne on the vesicles of conidiophores, forming dense, columnar conidial heads [43]. The molecular studies based on the nucleotide sequence of the ITS region revealed that the thermophilic fungal isolate VP2T showed a high degree of sequence similarity (>95%) with *A. fumigatus* strain F1 (MF276893.1) (at E = 0.0). The dendrogram constructed using nucleotide sequences of VP2T and other strains of *A. fumigatus* (derived from NCBI) showed that VP2T forms a separate clade with *A. fumigatus* isolate SF8 (KX011021.1), indicating VP2T to be a recently evolved novel strain of *A. fumigatus* based on their respective branch length analysis (Figure 2).

### 3.2. Analyzing the Whole Genome Sequence of the Isolated A. fumigatus Strain VP2T

The *A. fumigatus* strain VP2T genome was sequenced employing next-generation Illumina sequencing technology. To ensure comprehensive coverage and accuracy, a paired-end (PE) library with an average insert size of 500 base pairs was prepared using the highly efficient Illumina HiSeq 4000 platform. Subsequently, this process resulted in 2.91 Gb of clean, short-sequence PE reads, displaying an average read length of 151 base pairs. The resulting assembly (generated using Masurca v4.1.0) of the nuclear genome comprised 5702 scaffolds, with 3034 supercontigs larger than 1 kb in size, alongside 1159 supercontigs exceeding 5 kb (Table 1). The whole genome sequence FASTA file is accessible on NCBI website with the Accession: PRJNA1302681.

These scaffolds were systematically structured to ensure optimal sequencing integrity, with an N50 scaffold length of 14,646 and an L50 scaffold count of 673, indicating robust genome assembly. The assembly statistics revealed a gap of 0.072%. Moreover, analysis of the sequenced *A. fumigatus* genome unveiled a G-C content of 49.04%. The Repeat analysis performed using RepeatMasker (v4.1.1), and RepeatModeler (v2.0.1) revealed 3.82% (~1.2 Mb) of masked bases. These consisted of 427 retroelements (0.49 Mb), 257 DNA transposons (0.18 Mb), a significant 1.01 Mb (3.17%) of total interspersed repeats, and 0.17 Mb (0.55%) of simple repeats.

The completeness of the *A. fumigatus* genome was evaluated through Benchmarking Universal Single-copy Orthologs (BUSCO) analysis. The Fungi_odb10 taxonomic lineage, which consists of 758 total BUSCO groups, provided a comprehensive benchmark for comparison. Remarkably, the analysis revealed high levels of completeness, with 93.3% of the expected BUSCO groups identified as complete (C) within the genome. Additionally, only a minimal fraction of genes were found to be fragmented (F), accounting for 4.7% of the total BUSCO groups. Moreover, a mere 2.0% of BUSCO groups were classified as missing (M), indicating a remarkably thorough coverage of the genome.

Gene prediction conducted through the state-of-the-art AUGUSTUS (v.3.1.0) software uncovered a total of 12,675 predicted genes. Additionally, annotation against the UniProt database revealed 10,521 annotated genes, providing valuable insights into the functional repertoire encoded within the genome.

### 3.3. Biomass Degrading Machinery in A. fumigates Strain VP2T

The genome of *A. fumigatus* VP2T encodes a robust and diverse repertoire of lignocellulolytic enzymes, enabling the efficient degradation of plant biomass. Although the *A. fumigatus* VP2T genome assembly consists of multiple scaffolds (5702 scaffolds; N50 = 14.6 kb), BUSCO analysis indicated a high level of completeness (93.3% complete BUSCOs), suggesting that the majority of coding genes are represented in the assembly. The identification of carbohydrate-active enzymes (CAZymes) in this study was performed using conserved catalytic domain-based annotation approaches, which allow reliable gene prediction even when genome assemblies are fragmented. Nevertheless, the fragmented nature of the assembly may limit precise reconstruction of large CAZyme gene clusters and their genomic organization. Therefore, the present analysis primarily focuses on the identification and functional categorization of individual CAZyme genes rather than detailed cluster-level synteny. Future improvements using long-read sequencing technologies are expected to resolve genome contiguity and enable deeper insights into CAZyme gene cluster architecture and co-regulation.

**Figure 3:**
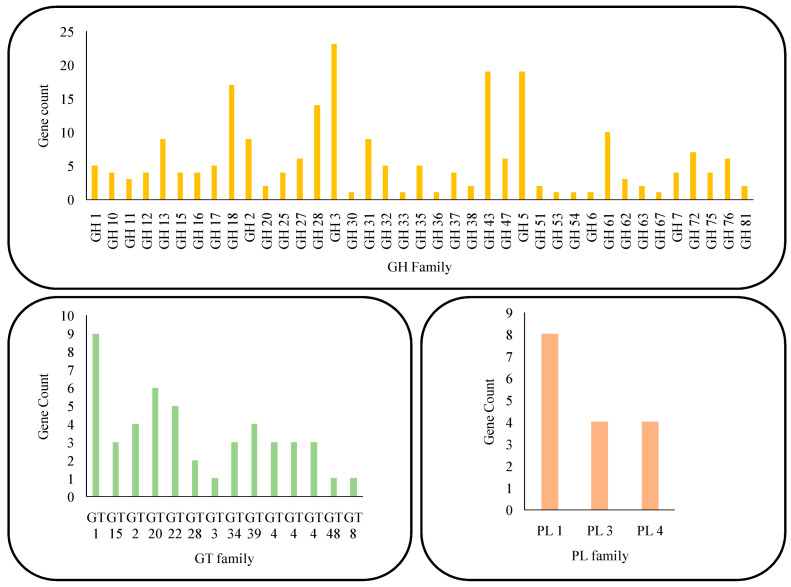
The CAZY enzymes present in the genome of *A. fumigatus* strain VP2T.

The identified genes were categorized based on their functional roles in cellulose and hemicellulose degradation, and auxiliary and accessory enzymatic activities (Figure 3 and Table 2). Key cellulolytic enzymes include endoglucanases (GH61/AA9), cellobiohydrolases (GH7), and β-glucosidases (GH3), which collectively dismantle cellulose into fermentable sugars [44]. Hemicellulose degradation is facilitated by a suite of enzymes such as endoxylanases (GH10), β-xylosidases (GH43), and β-mannanases (GH5), highlighting the fungus’s ability to access complex polysaccharides in lignocellulose [45].

**Table 2:** The CAZY enzymes present in the genome of *A. fumigatus* VP2T.

Enzymes	E.C. Number	Gene Name	Family
Cellulases			
Endoglucanase	3.2.1.4	NA	GH61 (AA9)
Cellobiohydrolase	3.2.1.91	NA	GH 7
β-glucosidase	3.2.1.21	NA	GH 3
α-glucosidase	3.2.1.20	NA	GH 31
Hemicellulases			
Endoxylanase	3.2.1.8	NA	GH10
β-xylosidase	3.2.1.37	*XlnD*	GH 43
α-glucuronidase	3.2.1.139	NA	GH 67
Endo-arabinase	3.2.1.99	NA	GH 43
β-mannanase	3.2.1.25	NA	GH 5
β-mannosidase	3.2.1.78	NA	GH 5
Auxiliary enzymes			
Cellobiose dehydrogenase	1.1.99.18	NA	GMC oxidoreductase
LPMOs	1.14.99.53–56	Multiple genes predicted (*n* = 3)	AA9
Laccase	1.10.3.2	*TilA*	Multicopper oxidase
Accessory Enzymes			
Endo-1,4-beta-mannosidase F	3.2.1.78	*manF*	GH5
Chitin synthase C	2.4.1.16	*chsC*	GT2
pectate lyase C	4.2.2.2	*plyC*	PL1
glycosidase crf1	3.2.-.-	*crf1*	GH16
Endo-1,4-beta-xylanase xynf11a	3.2.1.8	*xlnA*	GH11
Endochitinase B1	3.2.1.14	*chiB1*	GH18
1,3-beta-glucanosyltransferase gel2	2.4.1.-	*gel2*	GH72
beta-glucosidase sun1	3.2.1.-	*sun1*	GH132
alpha-1,3-glucan synthase	2.4.1.183	NA	GH13; GT5
mannosyltransferase	2.4.1.109	NA	GT39
alpha-galactosidase B	3.2.1.22	*aglB*	GH27
exo-1,4-beta-xylosidase xlnD	3.2.1.37	*xlnD*	GH3
Pyranose oxidase	1.1.3.10	*p2ox*	AA3
endo-1,3(4)-beta-glucanase	3.2.1.6	NA	GH16
chitinase	3.2.1.14	NA	CBM18; CBM50; GH18
Endochitinase B1	3.2.1.14	*chiB1*	GH18

NA: Not applicable.

Auxiliary enzymes like cellobiose dehydrogenase (GMC oxidoreductase) and laccase (TilA, a multicopper oxidase) likely play synergistic roles in oxidative cleavage and lignin modification. The presence of lytic polysaccharide monooxygenases (LPMOs), although not fully annotated, further supports the oxidative degradation potentials [46]. In addition, accessory enzymes such as endochitinases (GH18), glucanosyltransferases (GH72), and mannosyltransferases (GT39) contribute to remodeling and hydrolyzing structural polysaccharides and glycoproteins [47]. Collectively, this enzymatic arsenal underscores *A. fumigatus* VP2T as a potent biomass-degrading organism, capable of thriving in lignocellulose-rich environments and offering significant biotechnological potential for sustainable bioresource conversion. The identified genes (Figure 3) were grouped according to the functionalities they perform and are compiled in Table 2.

Genome annotation further identified three putative genes encoding AA9-family lytic polysaccharide monooxygenases (LPMOs). These copper-dependent oxidative enzymes are known to catalyze the oxidative cleavage of crystalline cellulose, thereby enhancing the accessibility of glycoside hydrolases during lignocellulose degradation. In the present study, these AA9 genes were predicted based on conserved catalytic domains and homology to previously characterized fungal LPMOs. Because detailed gene-level functional characterization and expression analysis were beyond the scope of this study, the AA9 enzymes were not individually listed in Table 2 and were instead grouped under auxiliary oxidative enzymes. Nevertheless, their presence strongly supports the proposed CDH–LPMO oxidative system contributing to the efficient hydrolysis of untreated biomass observed in strain VP2T.

### 3.4. Optimization of Growth Conditions for the Thermophilic Aspergillus fumigatus Strain VP2T

**Figure 4:**
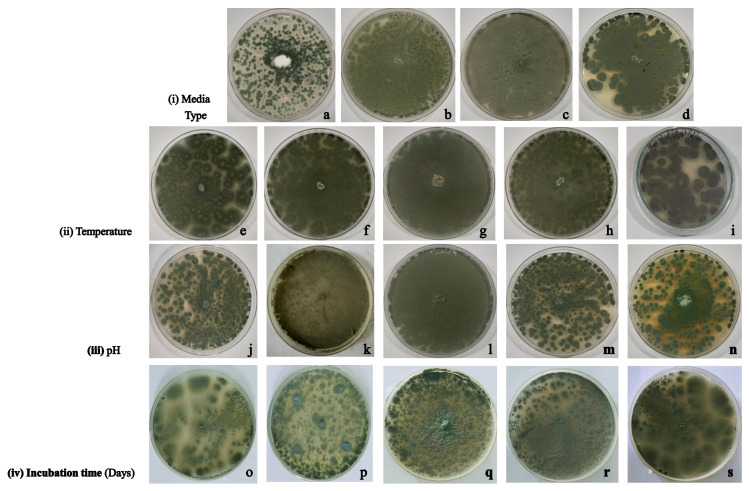
Optimizing the growth of *Aspergillus fumigatus* strain VP2T (i) on different media types: (**a**) Czapek yeast extract Agar, (**b**) Oatmeal Agar, (**c**) Potato Dextrose Agar, and (**d**) Yeast Phosphate soluble starch Agar; (ii) at different temperatures: (**e**) 35 °C, (**f**) 40 °C, (**g**) 45 °C, (**h**) 50 °C, and (**i**) 55 °C; (iii) at different pH: (**j**) 4.0, (**k**) 5.0, (**l**) 6.0, (**m**) 7.0 and (**n**) 8.0; and (iv) on different days of incubation: (**o**) Day 3, (**p**) Day 4, (**q**) Day 5, (**r**) Day 6 and (**s**) Day 7.

To determine the optimum conditions for the growth of Aspergillus fumigatus strain VP2T, several factors such as media types, temperature, pH, and incubation time were evaluated systematically (Figure 4). It was observed that VP2T showed good growth on all the selected media; however, PDA was able to support denser growth within 3 days from inoculation. Therefore, PDA was selected as the standard medium for further testing due to its ability to support consistent fungal development. The study on production of cellulases and xylanase by *A. fumigatus* SK1 also supports the use of PDA as a suitable medium for the growth of *A. fumigatus* [48]. The growth of VP2T strain was further assessed over a diverse range of temperatures (35–55 °C), demonstrating robust stability at the elevated temperatures with the most uniform and dense mycelial expansion occurring at 45 °C. The observed growth optimum of 45 °C distinguishes VP2T from typical mesophilic A. fumigatus isolates, usually growing at 37 °C optimally, aligning it with thermophilic strains optimized for industrial fermentation [22,49]. This elevated temperature profile is particularly advantageous for enzyme production at industrial scale, as it minimizes contamination risks and enhances the catalytic turnover rates of secreted cellulolytic enzymes. Finally, investigation of VP2T under diversified pH conditions indicated that the fungus remains viable across a broad range of pH 4.0–8.0. The observed growth pattern is in agreement with the literature reports on the pH range suitable for the growth of *A. fumigatus* [49]. Under acidic conditions (pH 4.0–6.0), *A. fumigatus* strain VP2T showed uniform growth pattern with its peak growth density at pH 6.0. It was also observed that the pH range of 7.0–8.0 leads to an increasingly patchy and restricted growth of VP2T.

### 3.5. Screening of the Lignocellulolytic Enzyme Activities in the Isolated Thermophilic Aspergillus fumigatus Strain VP2T

When cultured on cellulose, wheat bran, and rice straw (3:1:1) based (CWR) production medium, *A. fumigatus* strain VP2T was found to be a prolific producer of cellulases exhibiting 33.30 U/mL (≈666 U/gdS) endoglucanase, 3.73 U/mL (≈74.6 U/gdS) β-glucosidase, 0.48 U/mL (≈9.6 U/gdS) FPase, 0.55 U/mL (≈11.0 U/gdS) Avicelase, and 131.02 U/mL (≈2620.4 U/gdS) cellobiose dehydrogenase activities. In addition, the culture also showed low cellobiohydrolase and xylanase activities corresponding to 0.02 U/mL (≈0.4 U/gdS) and 14.22 U/mL (≈282.4 U/gdS), respectively. The enzymatic profile of *A. fumigatus* strain VP2T reveals a dynamic interplay of cellulolytic and auxiliary enzymes tailored for efficient lignocellulose degradation. The observed activities highlight a synergistic strategy for substrate utilization, balancing hydrolytic and oxidative mechanisms to maximize cellulose degradation [50,51]. The remarkably high endoglucanase activity highlights the strain’s proficiency in initiating cellulose hydrolysis by cleaving internal β-1,4-glycosidic bonds, generating oligosaccharides and reducing ends [44]. This is complemented by extraordinary CDH activity, which oxidizes cellobiose to cellobionolactone and cellobionic acid subsequently, mitigating product inhibition of β-glucosidase [52]. CDH also synergizes with lytic polysaccharide monooxygenases (LPMOs) to disrupt crystalline cellulose via oxidative cleavage [53,54]. This CDH-driven oxidative mechanism explains the relatively low FPase activity, as dependence on traditional exoglucanase-mediated processivity is reduced by oxidative disruption of cellulose microfibrils. Whole genome analysis of *A. fumigatus VP2T* (Section 3.2) reveals the presence of LPMO (AA9) genes, known for their activity against crystalline cellulose substrates. Coupled with the considerable enzymatic activity against Avicel, this strongly suggests the potential of the VP2T strain for LPMO expression in the fungus [55]. The detectable avicelase activity contrasts with negligible cellobiohydrolase, indicating a preference for paracrystalline cellulose (Avicel) over highly ordered crystalline regions. This aligns with the dominance of endoglucanase, which generates amorphous regions for CDH-mediated oxidative attack rather than the classical exoglucanase-driven processivity [56].

Interestingly, many studies on cellulases derived from *A. fumigatus* identify solid-state fermentation (SSF) as the predominant production method. Consequently, enzyme activities in these reports are typically expressed in units per gram of dry substrate (U/gDS). To facilitate direct comparison with existing literature, the enzymatic activities of *A. fumigatus* strain VP2T (SmF) were standardized and converted to equivalent U/gDS values as well. Table 3 and Table 4 present a comparative analysis of the enzymatic activity profiles between strain VP2T (wild type) and other *A. fumigatus* strains (wild type and recombinant/genetically improved) documented in prior studies. *A. fumigatus* strain VP2T, a wild-type strain, demonstrates a unique enzymatic profile that distinguishes it from both wild-type and recombinant counterparts under comparison. Its endoglucanase activity (33.2 IU/mL; 666 U/g) surpasses most strains, including *A. fumigatus* N2 (5.61 IU/mL) and JCM 10253 (19.5 IU/mL), positioning it as a potent degrader of amorphous cellulose [57,58]. While recombinant strains like *A. fumigatus* Af1 (65 IU/mL CMC) achieve higher hydrolytic outputs under optimized conditions [59], VP2T’s performance is remarkable for an unmodified organism under unoptimized conditions, reflecting its natural evolutionary refinement. The unparalleled feature of the *A. fumigatus* strain VP2T is its cellobiose dehydrogenase (DCIP) activity (131 IU/mL; 2620.4 U/g), which is lacking in all strains compared in Table 3 and Table 4. This oxidative prowess enables VP2T to synergize with lytic polysaccharide monooxygenases (LPMOs), disrupting crystalline cellulose through redox mechanisms, a trait rarely matched even in engineered strains like *A. fumigatus* AfAA9_B (8.33 U/g) [60].

However, VP2T lags in exoglucanase (0.48 IU/mL Filter Paper activity; 9.6 U/g) and xylanase (14.22 IU/mL; 282.4 U/g) activities compared to specialized strains. For instance, *A. fumigatus* Z5 achieves 144.6 U/g Filter Paper activity [68], and *A. fumigatus* AMA produces 2782 U/g xylanase [69]. These gaps reflect ecological adaptations and specialization of VP2T rather than its inherent deficiencies, as fungi rarely optimize all enzymatic activities simultaneously in nature, and instead, evolve niche-specific strategies.

Further, the enzymatic performance of *A. fumigatus* VP2T surpasses the commercial cellulase blend Cellic CTec2 in key activities critical for lignocellulose degradation, including endoglucanase (EG), β-glucosidase (βG), Avicelase, cellobiose dehydrogenase (CDH), and FPase (Table 5). This superiority suggests that VP2T’s tailored enzymatic arsenal efficiently addresses bottlenecks in cellulose hydrolysis, thus positioning VP2T as a promising, self-sufficient candidate for industrial biomass conversion, reducing dependency on multi-enzyme cocktails.

Conclusively, *A. fumigatus* strain VP2T demonstrates exceptional potential as a biocatalyst for cellulose-rich biomass conversion, owing to its unparalleled oxidative capacity and robust endoglucanase efficiency. To address low exoglucanase and xylanase outputs, the fungus can be subjected to gene editing, non-GMO strain improvement approaches, or adaptive evolution to enhance its versatility.

### 3.6. Evaluating and Comparing the Hydrolytic Potential of A. fumigatus Strain VP2T with Commercially Available Cellic CTec2 for Hydrolysis of Untreated and Pretreated Biomass

The hydrolytic efficiency of secretome derived from *A. fumigatus* strain VP2T was evaluated on untreated, alkali-pretreated, and acid-pretreated rice straw (RS), wheat straw (WS), and sugarcane bagasse (SB). The untreated substrates yielded 6.625 mg/mL (RS), 11.75 mg/mL (WS), and 10 mg/mL (SB) of total reducing sugars, whereas alkali pretreatment significantly enhanced sugar release, with RS, WS, and SB producing 20 mg/mL, 13.25 mg/mL, and 17.25 mg/mL, respectively. In addition, acid pretreatment resulted in intermediate yields of 8.75 mg/mL (RS), 7.5 mg/mL (WS), and 13.25 mg/mL (SB) after hydrolysis. Further, the hydrolytic potential of *A. fumigatus* strain VP2T was compared with the commercially available cellulase preparation Cellic CTec2. Strikingly, VP2T outperformed Cellic CTec2 on untreated rice straw (UTRS), generating 1.89-fold higher reducing sugars (Table 6). This aligns with VP2T’s enzymatic profile, where endoglucanase (EG) dominates by cleaving internal β-1,4-glycosidic bonds to generate amorphous cellulose regions, a mechanism recently validated in *Aspergillus* species [44]. Concurrently, CDH oxidizes cellobiose, activating LPMOs to disrupt crystalline cellulose, a synergistic process critical for untreated biomass degradation [52]. This oxidative–hydrolytic interplay is particularly advantageous for raw lignocellulose, where lignin and hemicellulose matrices impede classical enzymatic access. However, VP2T showed parity with CTec2 on untreated wheat straw (UTWS) and sugarcane bagasse (UTSB) and underperformed on alkali/acid-pretreated substrates. This may be attributed to the fact that pretreatment exposes crystalline cellulose, necessitating robust cellobiohydrolase (CBH) activity for processive hydrolysis, as demonstrated in modern biomass conversion studies [74]. VP2T’s negligible CBH activity (0.4 U/gDS) limits its efficiency here, unlike Cellic CTec2, which integrates CBH, LPMO, and β-glucosidase for balanced degradation.

Conclusively, 1.89-fold superiority on UTRS positions VP2T as a promising candidate for low-input bioprocessing of raw agricultural residues, reducing reliance on costly pretreatments. However, its limited CBH activity suggests that hybrid formulations combining VP2T’s secretome with exogenous exoglucanases could unlock broader applicability across pretreated and crystalline substrates.

While *A. fumigatus* VP2T demonstrates substantial industrial promise, it belongs to a species recognized as an opportunistic pathogen in immunocompromised individuals. However, *A. fumigatus* has a long history of cellulolytic applications, with multiple strains including ABK9 (cellulase/xylanase production), SK1 (oil palm cellulases), and A4112 (effluent hydrolysis) successfully deployed for enzyme production and biorefinery applications without biosafety concerns. Therefore, VP2T can be safely employed for large-scale enzyme production following similar protocols. Targeted genetic modifications of VP2T, including sporulation knockout of *flbA* and *brlA* genes to eliminate infectious spores and toxin biosynthesis disruption of the *gliP* cluster and *aflR* gene to prevent mycotoxin production, combined with standard containment procedures such as closed submerged fermentation to prevent aerosol and spore generation and HEPA-filtered air systems, can be adopted for seamless industrial bioprocesses.

### 3.7. Structural Analysis of Rice Straw Before and After Hydrolysis with Secretome Derived from A. fumigatus sp. Strain VP2T

**Figure 5:**
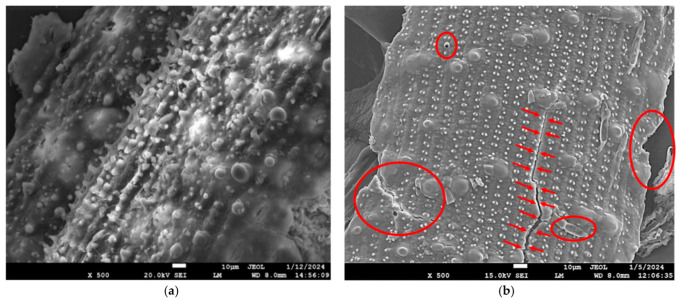
Scanning Electron Micrographs (SEM) of rice straw, showing (**a**) untreated straw with a dense, rigid morphology and well-preserved lignocellulosic fibers, and (**b**) VP2T secretome-treated rice straw exhibiting hallmarks of enzymatic hydrolysis including increased porosity, surface erosion, and fiber disintegration.

Scanning electron microscopy (SEM) analysis of untreated and *Aspergillus fumigatus* strain VP2T’s secretome-treated rice straw revealed distinct structural alterations critical to understanding the enzymatic degradation mechanism. Untreated rice straw (Figure 5a) displayed a smooth, intact surface with tightly packed fibres, a characteristic of the native lignocellulosic matrix where lignin and hemicellulose shield cellulose microfibrils [75]. This structural integrity explains the inherent recalcitrance of untreated biomass to enzymatic hydrolysis. In contrast, VP2T-treated rice straw (Figure 5b) exhibited pronounced surface degradation, including roughness, porosity, and exposed cellulose microfibrils. These morphological changes, such as cracks, pits, and fragmented fibres, align with VP2T’s enzymatic profile, dominated by endoglucanase (EG) and cellobiose dehydrogenase (CDH), which synergizes with lytic polysaccharide monooxygenases (LPMOs) to oxidatively disrupt crystalline cellulose and lignin-hemicellulose complexes [76].

## 4. Conclusions

This study presents a comprehensive characterization of *Aspergillus fumigatus* strain VP2T as a promising lignocellulose-degrading fungal system with potential applications in industrial biorefinery processes. Through an integrated approach combining genome analysis, enzymatic profiling, and biomass hydrolysis assays, the strain was shown to possess a diverse repertoire of carbohydrate-active enzymes supporting both hydrolytic and oxidative mechanisms of lignocellulose degradation. Notably, the enzymatic system of VP2T appears to rely strongly on oxidative strategies involving cellobiose dehydrogenase and predicted AA9-family lytic polysaccharide monooxygenases, which together enhance accessibility to cellulose within untreated biomass. This oxidative–hydrolytic synergy highlights an alternative biomass deconstruction strategy that may reduce the dependency on extensive chemical pretreatment typically required in conventional lignocellulosic bioconversion processes. The genomic and enzymatic features identified in this study position VP2T as a valuable candidate for the development of next-generation fungal enzyme systems and tailored enzyme cocktails for sustainable biomass conversion. Future work involving improved genome assemblies, enzyme characterization, and strain optimization will further elucidate the regulatory and structural basis of its lignocellulolytic machinery and facilitate its translation into industrial bioprocessing applications.

## Figures and Tables

**Table 1:** Genome statistics overview.

**G** **enome Assembly Statistics**	
Genome size (bp)	32,104,026
G + C content (%)	49.04%
Number of scaffolds	5702
Number of contigs	5787
Longest scaffold (bp)	83,381
Shortest scaffold (bp)	300
Number of scaffolds > 1 K nt (%)	3034 (53.2%)
Number of scaffolds > 10 K nt (%)	1159 (20.3%)
Mean scaffold size (bp)	5630
Median scaffold size (bp)	1755
N50 scaffold length (bp)	14,646
L50 scaffold count	673
A-T-G-C-N (%)	25.06%-24.93%-24.99%-24.96%-0.07%
Percent Gaps	0.072
**BUSCO Analysis (** ***n*** ** = 758; Lineage: Fungi_odb10)**	
Complete and single-copy BUSCOs	694
Complete and duplicated BUSCOs	13
Fragmented BUSCOs	36
Missing BUSCOs	15
**Gene Prediction and Annotation**	
Predicted genes	12,675
Annotated genes	10,521
**Repeat Analysis Statistics**	
Masked Bases (bp)	1,225,535 (3.82%)
Retroelements (number of elements)	427 (490,347 bp)
LTR elements	427 (490,347 bp)
Ty1/Copia	121 (107,106 bp)
Gypsy/DIRS1	306 (383,241 bp)
DNA transposons	257 (185,392 bp)
Tc1-IS630-Pogo	247 (179,654 bp)
Unclassified	868 (342,650 bp)
Total interspersed repeats	1,018,389 bp
Simple repeats	4207 (177,175 bp)
Low complexity	597 (29,971 bp)

**Table 3:** Comparison of cellulase and xylanase production by *A. fumigatus* strain VP2T with various *A. fumigatus* strains reported in the literature under shake flask conditions.

Activity Against Different Substrates (U/mL)										
S. NO.	Fungal Strain	TYPE	CMC	pNPL	pNPG	Avicel	DCIP	Filter Paper	Xylan	References
1.	*A. fumigatus VP2T*	Wild-type	33.2	0.02	3.73	0.55	131	0.48	14.22	This study
2.	*A. fumigatus N2*	Wild-type	5.61	ND	ND	ND	ND	ND	91.9	Lin et al. [57]
3.	*A. fumigatus AF293*	Wild-type	0.032	ND	ND	ND	ND	ND	10.82	de Gouvêa et al. [60]
4.	*A. fumigatus Af1*	Developed	65	ND	ND	4.7	ND	ND	ND	Alotaibi et al. [59]
5.	*A. fumigatus NITDGPKA3*	Procured	6.53	ND	80.1	ND	ND	1.02	193.58	Sarkar et al. [61]
6.	*A. fumigatus* *JCM 10253*	Wild-type	19.5	ND	0.083	ND	ND	ND	ND	Saroj et al. [58]
7.	*A. fumigatus*	Wild-type	ND	ND	0.104	ND	ND	0.0089	ND	Ximenes et al. [62]
8.	*A. fumigatus*	Wild-type	0.225	ND	0.085	ND	ND	ND	ND	Dahot and Noomrio et al. [63]
9.	*A. fumigatus*	Procured	ND	ND	ND	ND	ND	ND	531	Nair et al. [64]
10.	*A. fumigatus* *SBS58*	Wild-type	ND	ND	ND	ND	ND	35	38	Nair et al. [64]
11.	*A. fumigatus* *AR1*	Wild-type	ND	ND	ND	ND	ND	ND	228	Anthony et al. [65]
12.	*A. fumigatus* *SBS62*	Wild-type	ND	ND	ND	ND	ND	32	38	Nair et al. [64]
13.	*A. fumigatus SBS63*	Wild-type	ND	ND	ND	ND	ND	20	20	Nair et al. [64]
14.	*A. fumigatus* *FBSPE-05*	Wild-type	0.365	ND	ND	ND	ND	ND	ND	Grigorevski et al. [66]

*p*-nitrophenol-β-D-lactopyranoside (pNPL), *p*-nitrophenol-β-D-glucoside (pNPG), 2,6-dichlorophenol indophenol (DCIP) and Carboxymethyl cellulose (CMC).

**Table 4:** Comparison of cellulase and xylanase production by *A. fumigatus* strain VP2T (under SmF normalized to U/gDS) with various *A. fumigatus* strains reported in the literature under solid-state conditions.

Activity Against Different Substrates (U/gDS)										
S. NO.	Fungal Strain	Type	CMC	pNPL	pNPG	Avicel	DCIP	Filter Paper	Xylan	References
1.	*A. fumigatus VP2T*	Wild-type	664	0.4	74.6	11	2620.4	9.6	282.4	This study
2.	*A. fumigatus SBC4*	Wild-type	ND	ND	54	ND	ND	ND	573	Santos et al. [67]
3.	*A. fumigatus AfAA9_B*	Developed	ND	ND	ND	8.33	ND	ND	ND	de Gouvêa et al. [60]
4.	*A. fumigatus Z5*	Wild-type	526.3	ND	ND	ND	ND	144.6	ND	Liu et al. [68]
5.	*A. fumigatus AMA*	Wild-type	98.5	ND	250	ND	ND	4.0	2782	Soni et al. [69]
6.	*A. fumigatus*	Wild-type	14.71	ND	8.51	ND	ND	ND	42.7	Sherief et al. [70]
7.	*A. fumigatus*	Wild-type	240.2	ND	ND	ND	ND	9.73	2800	Soni et al. [71]
8.	*A. fumigatus KSA-2*	Wild-type	ND	ND	ND	ND	ND	ND	66	Ameen and Fuad et al. [72]
9.	*A. fumigatus*	Wild-type	5.54	ND	ND	ND	ND	0.288	ND	Gilna and Khaleel [73]

*p*-nitrophenol-β-D-lactopyranoside (pNPL), *p*-nitrophenol-β-D-glucoside (pNPG), 2,6-dichlorophenol indophenol (DCIP) and Carboxymethyl cellulose (CMC).

**Table 5:** Comparative analysis of the enzymatic profiles of *A. fumigatus* strain VP2T with commercial cellulase preparation Cellic CTec2.

Activity Against Different Substrates Under SmF (U/mL)								
Enzyme	CMC	pNPC	pNPL	pNPG	Avicel	DCPIP	Filter Paper	Xylan
*A. fumigatus* VP2T secretome	33.2 ± 0.71	0.527 ± 0.03	0.245 ± 0.02	3.73 ± 0.22	0.55 ± 0.12	131 ± 5.61	0.48 ± 0.16	14.22 ± 2.76
Cellic CTec2	30.78 ± 1.23	0.541 ± 0.07	0.25 ± 0.06	0.558 ± 0.09	0.334 ± 0.11	7.41 ± 1.91	0.233 ± 0.08	35.34 ± 3.87

*p*-nitrophenol-β-D-lactopyranoside (pNPL), *p*-nitrophenol-β-D-glucoside (pNPG), *p*-nitrophenol-β-Dcellobioside (pNPC), 2,6-dichlorophenol indophenol (DCIP) and Carboxymethyl cellulose (CMC). ±SE from triplicate experiments (*n* = 3) at 5% significance level (*p* < 0.05).

**Table 6:** Comparative analysis of the hydrolytic potential of *A. fumigatus* strain VP2T and commercial cellulase preparation Cellic CTec2.

Reducing Sugars Released from Different Substrates (mg/mL)									
Sample	UTRS	ALRS	ACRS	UTWS	ALWS	ACWS	UTSB	ALSB	ACSB
*A. fumigatus* VP2T secretome	6.625 ± 0.23	20 ± 1.35	8.75 ± 0.87	11.75 + 1.68	13.25 ± 1.93	7.5 ± 0.34	10 ± 1.21	7.5 ± 1.23	13.25 ± 1.19
Cellic CTec2	3.5 ± 0.46	40 ± 2.63	25 ± 2.11	12.5 ± 1.98	35 ± 2.71	13.25 ± 1.48	15 ± 2.01	43 ± 3.26	15 ± 1.85

Untreated (UT), alkali-treated (AL), and acid-treated (AC) Rice straw (RS), wheat straw (WS), and sugarcane bagasse (SB) were added as 7% of the substrate loading rate. A protein loading rate of 10 mg/gDS was used to commence the hydrolysis reaction The final volume of the reaction was adjusted to 1 mL with sodium acetate buffer (50 mM; pH 4.8) and incubated at 50 °C for 72 h. ±SE from triplicate experiments (*n* = 3) at 5% significance level (*p* < 0.05).

## Data Availability

The whole genome sequence FASTA file is accessible on NCBI website (https://www.ncbi.nlm.nih.gov/bioproject/PRJNA1302681/) with the Accession: PRJNA1302681.
